# Expanding clinical spectrum of PAICS deficiency: Comprehensive analysis of two sibling cases

**DOI:** 10.1038/s41431-024-01752-2

**Published:** 2024-11-27

**Authors:** Wen-Chin Weng, Vaclava Skopova, Veronika Baresova, Yao-Lin Liu, Hsueh-Wen Hsueh, Yin-Hsiu Chien, Wuh-Liang Hwu, Olga Souckova, Ales Hnizda, Stanislav Kmoch, Ni-Chung Lee, Marie Zikanova

**Affiliations:** 1 Department of Pediatrics, National Taiwan University Hospital, Taipei, Taiwan; 2 Department of Pediatrics, National Taiwan University College of Medicine, Taipei, Taiwan; 3 Department of Paediatrics and Inherited Metabolic Disorders, First Faculty of Medicine, Charles University and General University Hospital in Prague, Prague, Czech Republic; 4 Department of Ophthalmology, College of Medicine, National Taiwan University Hospital, National Taiwan University, Taipei, Taiwan; 5 Department of Neurology, National Taiwan University Hospital, Taipei, Taiwan; 6 Department of Medical Genetics, National Taiwan University Hospital, Taipei, Taiwan; 7 OMICS Mass Spectrometry Core Facility, Biology Departments, Faculty of Science, Charles University, BIOCEV, Vestec, Czech Republic

**Keywords:** Neurodevelopmental disorders, Metabolic disorders, Genetic counselling

## Abstract

De novo synthesis of purines (DNPS) is a biochemical pathway that provides the purine bases for synthesis of essential biomolecules such as nucleic acids, energy transfer molecules, signaling molecules and various cofactors. Inborn errors of DNPS enzymes present with a wide spectrum of neurodevelopmental and neuromuscular abnormalities and accumulation of characteristic metabolic intermediates of the DNPS in body fluids and tissues. In this study, we present the second case of PAICS deficiency due to bi-allelic variants of *PAICS* gene encoding for a missense p.Ser179Pro and truncated p.Arg403Ter forms of the PAICS proteins. Two affected individuals were born at term after an uncomplicated pregnancy and delivery and presented later in life with progressive cerebral atrophy, epileptic encephalopathy, psychomotor retardation, and retinopathy. Plasma and urinary concentrations of dephosphorylated substrates of PAICS, AIr and CAIr were elevated, though they remained undetectable in skin fibroblasts. Both variants affect structural domains in SAICARs catalytic site and the oligomerization interface. In silico modeling predicted negative effects on PAICS oligomerization, enzyme stability and enzymatic activity. Consistent with these findings, affected skin fibroblasts were devoid of PAICS protein and enzyme activity. This was accompanied by alterations in contents of other DNPS proteins, which had co-localized in granular structures that are characteristic of purinosome formation. Our observation expands the clinical spectrum of PAICS deficiency from recurrent abortions and fatal neonatal form to later onset neurodevelopmental disorders. The rarity of this condition may be based on poor clinical recognition and limited access to specialized laboratory tests diagnostic for PAICS deficiency.

## Introduction

**Fig. 1:**
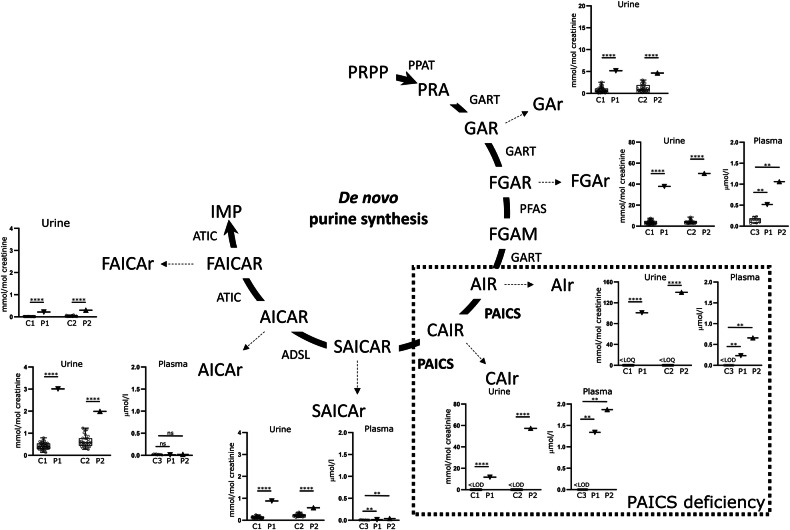
PAICS deficiency and accumulation de novo purine synthesis intermediates. Glycineamide riboside (GAr), formylglycineamide riboside (FGAr), aminoimidazole riboside (AIr), carboxyaminoimidazole riboside (CAIr), sukcinylaminoimidazolecarboxamide riboside (SAICAr), aminoimidazolecarboxamide riboside (AICAr) and formylaminoimidazolecarboxamide riboside (FAICAr) were quantified in the plasma and urine of patients. Concentrations of all DNPS intermediates were significantly increased in patients´ urine compared to control samples. In plasma samples from affected individuals, concentrations of upstream intermediates were also significantly increased compared to control samples. Concentrations are shown in standardized boxplot graphs with box from the first to third quartiles, whiskers to minimum and maximum, and all data points are displayed (open circles for controls and triangles for patients). C1 denotes urine controls aged 6–9 years (*n* = 47), C2 denotes urine controls aged 1–3 years (*n* = 33), and C3 denotes plasma controls aged less than 16 years (*n* = 9). P1 and P2 represent patient cases 1 and 2, respectively. The levels of GAr and FAICAr in plasma were below the limit of detection (LOD) and therefore not shown. If only control samples are bellow LOD, this is indicated on the graph by ‘<LOD’. One-sample Wilcoxon signed-rank tests were calculated by GraphPad Prism software and *p*-values are shown: **** for *P* < 0.0001, ** for *P* < 0.01, and ns for *P* ≥ 0.05.

De novo synthesis of purines (DNPS) is a biochemical pathway that provides the purine bases for synthesis of essential biomolecules such as nucleic acids, energy transfer molecules, signaling molecules and various cofactors. DNPS starts with the phosphoribosyl pyrophosphate (PRPP) and concludes with the production of inosine monophosphate (IMP), (Fig. 1). The process comprises a series of 10 enzymatic reactions and requires the coordinated action of six enzymes that upon cellular needs temporarily assemble in the cytoplasm into a liquid-like condensates called purinosomes [1, 2].

To date, inborn errors of three of DNPS enzymes have been identified: adenylosuccinate lyase (ADSL, EC 4.3.2.2) deficiency [OMIM 103050] [3] AICA-ribosiduria [OMIM 608688] [4] and aminoimidazole ribonucleotide carboxylase (AIRc, EC 4.1.1.21)/ phosphoribosylaminoimidazolesuccinocarboxamide synthase (SAICAIRs, EC 6.3.2.6) (PAICS) deficiency [OMIM 619859] [5]. Affected individuals presented with a wide spectrum of neurodevelopmental and neuromuscular abnormalities and accumulation of characteristic metabolic intermediates of the DNPS in body fluids and tissues [3, 6]. Biochemical screening of these diagnostic intermediates is only available in a few specialized laboratories [7, 8]. As a result, only about 100 cases of ADSL deficiency (www.adenylosuccinatelyasedeficiency.com), 6 cases of AICAribosiduria [6, 9, 10] and one case of PAICS deficiency [5] have been identified. Inborn errors of DNPS thus remain clinically poorly characterized, unrecognized and undiagnosed.

In this study, we present clinical, biochemical, molecular and cellular characteristics of two siblings identified with bi-allelic variants in the *PAICS* gene. Our investigation expands the clinical spectrum of PAICS deficiency and demonstrates for the first time the presence of specific metabolic intermediates in body fluids of affected individuals that are diagnostic for PAICS deficiency.

## Methods

### Participants and clinical examination

Two affected siblings were identified and received clinical assessments including laboratory examinations including blood samples, electroencephalograms, and brain magnetic resonance imaging. Skin punch biopsies were obtained under local anesthesia from the two siblings and skin fibroblasts were cultured following a standard protocol.

### Chemicals

Aminoimidazole ribotide (AIR) and all necessary commercially unavailable standards were prepared according to established methods [5, 11, 12]. Other metabolites were purchased from Toronto Research Chemicals (FAICAR, ^13^C_2_,^15^N-AICAr), Cambridge Isotope Laboratories (^13^C_5_-hypoxanthine), MedChemExpress (CAIR), Gibco, Invitrogen (DMEM/F12, FBS, DMEM) respectively. Sigma-Aldrich supplied the remaining reagents, except where noted differently.

### Sequencing analysis

Genomic DNA was isolated from venous blood using standard protocol. Whole exome sequencing (WES) and whole mitochondrial genome sequencing were performed and analyzed as previously described [13, 14]. The mtDNA sequence was aligned to a mitochondrial reference genome (NC_012920.1) using BWA MEM, followed by variant calling by the mtDNA Server Mutserve (v.2.0.0.1) [15].

### Structural impact of the identified variants

The effect of identified variants was assessed using available crystal structure of PAICS (PDB ID 2H31) [16]. Structural models were visualized using Pymol Viewer (Schrodinger).

Structural stability was assessed using the Rosetta Online Server (ROSIE 2) [17]. Structural models were modified in Pymol subsequently subjected to relaxation (Rosetta relax) and then evaluated using Score.

### Culturing and lysis of skin fibroblasts and peripheral blood mononuclear cells

The patients skin fibroblasts were maintained in the DMEM/F12 nutrition mix medium, supplemented with 10% FBS and 1% of Penicillin/Streptomycin (P/S) and 0.03 mM adenine (purine rich = PR). For purinosome formation detection, the cells were cultivated in the purine-depleted medium (PD): DMEM supplemented with 10% dialyzed FBS (dFBS) and 1% P/S, 48 h prior to the experiment [1]. 1.5 × 10^6^ SF cells were PBS-rinsed, centrifuged at 400x*g* for 5 min at 4 °C, resuspended in 30 μl lysis buffer (30 mM KH_2_PO_4_, pH 6.0, 0.5% polyethylene glycol ether W-1, Protease Inhibitor Cocktail Tablets), and kept on ice for 45 min. The sample was sonicated (4×5 s bursts), and centrifuged at 17000x*g* for 20 min at 4 °C. Peripheral blood mononuclear cells (PBMCs) were prepared according to the standard protocol and lysed in 50 μl of buffer A (10 mM Tris (pH 8.2), 2 mM EDTA, 10 mM KCl, 1 mM DTT, and 4% glycerol) by sonicating 4×15 s at 60% amplitude, and centrifuged at 17000 x *g* for 20 min at 4 °C.

### PAICS enzyme catalytic activity in cells

Protein levels in SF and PBMC lysates were measured using Bradford reagent. The activity of PAICS was assessed with the methods previously described [5]. PAICS activity in PBMCs was tested similarly with minor modifications: 2.5 mM aspartate, 1 mg/ml PBMC lysate, and 0.07 mM AIR were used, and the reaction mixture was incubated for 1 h. Succinylaminoimidazolecarboxyamide ribotide (SAICAR) and carboxyaminoimidazole ribotide (CAIR) detection was performed using LC-MS/MS after a two-fold dilution of the samples. Tests were conducted three times.

### Western blot (WB) analysis

Total protein contents were detected by No-Stain Protein Labeling Reagent (Thermo Fisher Scientific). Cell lysates were separated on 10% SDS-PAGE and proteins were transferred to a PVDF membrane. The membrane was blocked using 5% BSA and incubated with primary antibodies: anti-PPAT rabbit polyclonal (ARP46079, Aviva), anti-GART mouse monoclonal (H00002618-B01P, Abnova), anti-PFAS rabbit polyclonal (ARP46181, Aviva), anti-PAICS mouse monoclonal (TA501470, Origene), anti-ADSL rabbit polyclonal (HPA000525, Sigma), anti-ATIC mouse monoclonal (ab33520, Abcam), anti-HPRT rabbit polyclonal (GTX113466, GeneTex), anti-GAPDH mouse IgM monoclonal (G8795, Sigma), and anti-actin rabbit polyclonal (A2103, Sigma). Peroxidase-conjugated secondary antibodies goat anti-mouse IgG (Sigma) and IgM (Pierce) and goat anti-rabbit IgG (Thermo Fisher Scientific), respectively were used for detection. Chemiluminescence was achieved using Clarity or Clarity MAX Western ECL Substrate (Bio-Rad) and captured by ChemiDoc MP Imaging System (Bio-Rad) and normalization to No-Stain labeling was calculated by ImageLab software.

### LC-MS/MS analysis

3x volume of frozen 80% methanol was added to 200 µl of medium or 50 µl SF cell lysate with protein concentration 1 mg/ml or 50 µl of plasma and incubated overnight at −80 °C. The following day, the samples were centrifuged, and the supernatants were evaporated by speed-vac. Obtained pellets were dissolved in 50 µl of water. Urine samples were diluted to 0.1 mM creatinine. All samples were centrifuged briefly and 45 µl of supernatant was mixed with 5 µL of internal labeled standard mixture. 5 µL of the sample was injected into the LC-MS/MS system consisting of an Agilent 1290 Infinity LC System (Agilent Technologies) equipped with a Prontosil 120-3-C18 AQ column (150*3 mm, 3 µm, Bischoff) coupled with an API 4000 triple quadrupole mass spectrometer with an electron spray ionization operated by Analyst software (Applied Biosystems), as previously described [5, 11]. MS parameters are presented in Supplementary Table S1. For lysates and media, the SRMs were separated to time periods, for urine, plasma and repeated measurements only subset SRM for ribosides, CAIR, formylglycineamide ribotide (FGAR), inosine, hypoxanthine and internal standards were used. For activity assays, the subset of SRM for all ribotides, aminoimidazole riboside (AIr), carboxyaminoimidazole riboside (CAIr) and succinylaminoimidazolecarboxyamide riboside (SAICAr) was used.

### Stability assay

Urine samples of patients (with concentrations 5.1, and 0.9 mmol/L of creatinine, resp.) and control sample were aliquoted and stored in different storage conditions: −80 °C without any manipulation, −80 °C with 5 cycles of thawing and freezing, −20 ° for one month, 4 °C for one week, 25 °C for one day, 25 °C for three days. Then they were diluted to 0.1 mmol/L of creatinine and analyzed by LC-MS/MS.

### Cloning of mutated pMAL-PAICS and recombinant PAICS Proteins catalytic activity

*PAICS* variants were inserted into pMAL-PAICS_wt vector [5] using the GeneArt™ Site-Directed Mutagenesis System (Thermo Fisher Scientific) following conventional methods. Recombinant wt and *PAICS* variant proteins were produced in *E. coli* and purified via affinity chromatography, as previously described [18]. Their enzymatic activities were measured using 25 mg/L of the purified recombinant fusion proteins, following previously established protocols [5] and quantifying the product, SAICAR, using HPLC, and intermediate, carboxyaminoimidazole ribotide (CAIR), after diluting samples using LC-MS/MS.

### Blue native (BN) electrophoresis

MBP-PAICS recombinant proteins were cleaved by Factor Xa protease (NEB) for one and half hours in RT and then BN electrophoresis was performed as previously described [19]. Subsequently, the gel was incubated in 300 mM Tris-Cl, pH 8.6 with 1% SDS for one hour and then the proteins were transferred to PVDF membrane. Total proteins were detected by No-Stain Protein Labeling Reagent (Thermo Fisher Scientific) following the method of WB analysis described above. The assay was performed three times and the densitometry for octamer bands was calculated with ImageLab software (Bio-Rad).

### Immunofluorescence

For parallel immunodetection of phosphoribosyl pyrophosphate amidotransferase (PPAT); and glycinamide ribonucleotide synthetase/glycinamide ribonucleotide transformylase/aminoimidazole ribonucleotide synthetase (GART) or ADSL and GART the cells were incubated with specific antibodies. PPAT was detected with polyclonal rabbit anti-PPAT antibody (ARP46079, Aviva), diluted 1:50, GART was detected with monoclonal mouse anti-GART antibody (H00002618-B01P, Abnova) diluted 1:50, and ADSL was detected with polyclonal rabbit anti-ADSL antibody (HPA000525, Sigma) diluted 1:20, and incubated overnight at 4 °C.

Detection of bound primary antibodies was achieved using Donkey anti-Mouse IgG Alexa Fluor®488 and Goat anti-Rabbit IgG Alexa Fluor®555 secondary antibodies (Thermo Fisher Scientific) diluted 1:1000 in 5%BSA in PBS incubated 1 h at 37 °C. Slides were mounted in ProLong Gold Antifade Mountant with DAPI (Thermo Fisher Scientific) and analyzed by confocal microscopy.

### Image acquisition and analysis

XYZ images were sampled according to Nyquist criterion using a Leica SP8X laser scanning confocal microscope, HC PL Apo objective (633, N.A.1.40), 405 nm diode/50 mW DMOD Flexibl, and 488, 555 and 647 laser lines in 470–670 nm 80 MHz pulse continuum WLL2. Images were restored using a classic maximum likelihood restoration algorithm in the Huygens Professional Software (SVI, Hilversum, The Netherlands) [20]. The colocalization maps employing single pixel overlap coefficient values ranging from 0–1, were created in the Huygens Professional Software [21]. The resulting overlap coefficient values are presented as the pseudo color, which scale is shown in corresponding lookup tables (LUT).

## Results

### Clinical and genetic analyses

**Fig. 2:**
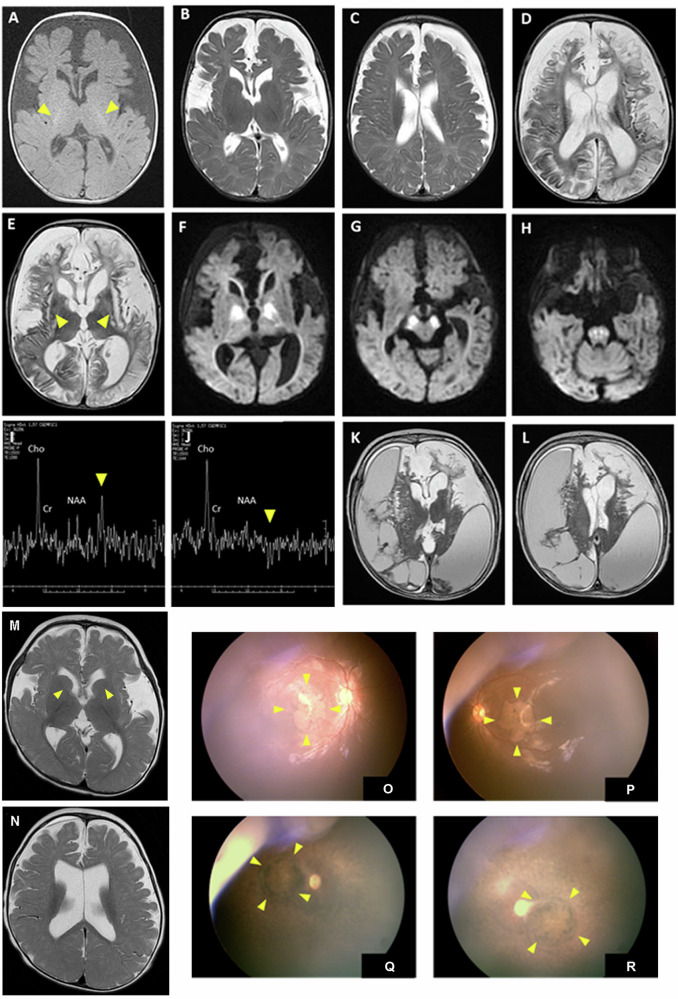
Brain imaging and ophthalmological findings in PAICS-deficient patients. **A**–**C** **Brain image changes in patient 1 axial MRI at 3 months of age**. Axial T1-weighted MRI image **A** revealed visible myelination of only the posterior limb of the internal capsules (arrowheads), enlargement of the ventricles, chronic subdural effusion predominantly in the frontotemporal region, and widened Sylvian fissures with failed operculization. These features were also evident in axial T2-weighted imaging **B**, **C**. **D**–**J** **Axial MRI and MRS in patient 1 at 7 months of age**. Axial T2-weighted imaging revealed more extensive cerebral atrophy and hyperintensities observed in both frontal, temporal areas and the putamen (arrowheads) **D**, **E**. Diffusion-weighted imaging (DWI) demonstrated restricted diffusion coefficients at both globus pallidum **F**, both cerebral peduncles **G**, and the central pons **H**. Magnetic resonance spectroscopy (MRS) of metabolites at long TE showed reduced NAA/Cre and NAA/Cho ratios, elevated Cho/Cre ratio, and the presence of lactate duplets (arrowheads) **I**, **J**. **K**, **L** **Axial MRI in patient 1 at 13 months of age**. Axial T2-weighted imaging of revealed rapid atrophy of cerebrum accompanied by notable subdural effusion. **M**, **N** **Brain image in patient 2 at the age of 1 year**. Axial T2-weighted imaging revealed poor myelination of the anterior limb of the internal capsules (arrowheads), enlargement of the ventricles, cerebral atrophy with frontotemporal predominance, and widened Sylvian fissures with failed operculization. **O**–**R** **Fundus photos of patient 2 at the age of one year and patient 1 at the age of 7 years**. The pictures showed significant retinal pigmentary changes and atrophy in the macular area in bilateral eyes, suggesting the presence of macular dystrophy **O**, **P** in patient 2. Similar but more pronounced and extensive features of the fundus were also observed in patient 1 at the age of 7 and a half years, implying a deteriorating disease course **Q**, **R**.

The index case (patient 1) was the first male child of non-consanguineous Taiwanese parents. He was born at term after an uncomplicated pregnancy and delivery. The infant presented with progressive poor appetite and irritability at 2 months. At 4 months of age, brain MRI revealed delayed or absent myelination, enlargement of the ventricles and chronic subdural effusion with frontotemporal predominance (Fig. 2A–C). Sylvian fissures were significantly widened and features of failed operculization were evident (Fig. 2A, B). The infant presented to the hospital at the age of 6 months due to a seizure attack and psychomotor regression, without achieving any motor milestone. Neurological examination revealed microcephaly, obvious truncal hypotonia, peripheral spasticity, hyper-reflexia and extensor plantar response in both lower extremities. The electroencephalogram showed diffuse slow waves, low background tracing and intermittent interictal focal epileptic discharges. Follow-up brain MRI scans at 7 months of age revealed more extensive cerebral atrophy and T2 hyperintensities observed in both frontal, temporal areas, as well as in the putamen (Fig. 2D, E). Diffusion-weighted imaging (DWI) demonstrated restricted diffusion coefficients of the central pons, both cerebral peduncles, and both globus pallidi (Fig. 2F–H). Brain magnetic resonance spectroscopy (MRS) of metabolites at long TE showed reduced NAA/Cre and NAA/Cho ratios, elevated Cho/Cre ratio, and the presence of lactate duplets (Fig. 2I, J). Fundoscopic examination showed macular dystrophy at the age of 7 months. Further evaluations including echocardiography, abdominal ultrasonography and metabolic surveys such as plasma amino acid and tandem mass spectrometry for acylcarnitine profiles yielded nonspecific findings.

Due to suspicion of mitochondrial disorders, a muscle biopsy of the quadriceps muscle was conducted at 7 months. Pathology indicated a few ragged-red fibers on Gomori-trichrome stain, but mitochondrial morphology did not exhibit abnormalities under electron transmission microscopy. Analysis of mitochondrial electron transfer chain activity revealed mitochondrial proliferation (citrate synthase activity was 136%), and respiratory chain activities were not deficient (46–74% of the control mean after adjusting for citrate synthase activity). Even with the prescription of ubiquinone, carnitine, and vitamin B, the infant continued to experience neuro-regression, leading to being bedridden, feeding difficulty requiring gastrostomy and lack of eye tracking. Follow-up brain MRI scans at 13 months of age showed rapid cerebral atrophy with massive subdural effusion (Fig. 2K, L). The boy died at the age of 8 years due to a severe infection leading to respiratory failure.

The younger sister of the index case (patient 2) was born at a gestational age of 38 weeks after an uneventful pregnancy when the brother was six and a half years old. She presented at 11 months with poor sucking ability, poor weight gain, developmental delay, and suspected focal epileptic seizures for one month. Neurological examination also revealed microcephaly, truncal hypotonia and peripheral hypertonia. On admission, frequent focal seizures with hand clonic movements lasting for seconds were observed and the EEG revealed frequent ictal or electrographic seizures primarily arising from the right temporal region. Interictal multifocal spikes and waves in the bilateral frontal and right temporal regions were also seen. Her seizures were gradually controlled with the initiation of levetiracetam and subsequently adding lacosamide. The brain MRI images at one year of age also indicated delayed or absence of myelination, ventricular enlargement, cerebral atrophy with frontotemporal predominance, and notably widened Sylvian fissures with features of failed operculization were evident (Fig. 2M, N), demonstrating a similar but less severe pattern compared to the brain MRI of her brother. Similar to her brother, evaluations including echocardiography, abdominal ultrasonography and metabolic surveys such as plasma amino acid and tandem mass spectrometry yielded nonspecific findings. Funduscopic examination revealed macular dystrophy at the age of one year (Fig. 2O, P). Similar features of the fundi were also observed in her brother at the age of 7 and a half years (Fig. 2Q, R). The patient is now 2 years old and is able to roll over, sit independently, stand with support, and grasp objects. She is unable to follow commands but can respond with a smile to voices or people, wave her hand, and enjoy listening to music. The frequency of seizures is approximately 2-3 times per month presenting with focal tonic seizures lasting for seconds, which are under control with the use of levetiracetam, lacosamide and clobazam.

Genetic analysis using SNP array and whole mt DNA sequencing of blood samples from both siblings did not identify any pathogenic variants. However, upon comparing the genetic profiles of the parents and siblings, WES detected the compound heterozygous variants *PAICS* NM_001079524.2 c.535 T > C (p.Ser179Pro) and c.1207 C > T (p.Arg403Ter) in both siblings. The c.535 T > C (p.Ser179Pro) variant was paternally inherited, while the c.1207 C > T (p.Arg403Ter) variant was maternally inherited. Neither variant was reported in the ClinVar database. Missense substitution p.Ser179Pro, detected in trans with the p.Arg403Ter nonsense stop codon, was predicted as damaging by seven of 13 bioinformatic tools and evaluated according to the ACMG guidelines as likely pathogenic (PM2, PM3, PP3, PP4). The p.Arg403Ter was predicted as deleterious by several bioinformatic tools (LRT, CADD, and FATHMM-MKL) and evaluated according to the ACMG guidelines as pathogenic (PVS1, PM2, PM3, PP4).

The clinical presentations of all reported cases of PAICS deficiency are summarized in Supplementary Table S2.

### Metabolites analysis

To assess biochemical consequences of identified *PAICS* variants, we used quantitative liquid chromatography–tandem mass spectrometry (LC-MS/MS) and analyzed presence of the DNPS intermediates in plasma, urine and skin fibroblasts.

In plasma samples from affected individuals, we detected an abnormal presence of dephosphorylated substrates of PAICS, AIr and CAIr, at ±1 μM concentration. The concentration of formylglycineamide riboside (FGAr), a dephosphorylated substrate of PFAS, was increased at a similar ±1 μM concentration. Concentrations of SAICAr and AICAr were in control range, glycineamide riboside (GAr), phosphoribosylformylglycinamidine (FGAM) and formylaminoimidazolecarboxamide riboside (FAICAr) were under limit of quantification.

In urine samples from affected individuals, we detected the abnormal concentrations of all DNPS intermediates. Their concentrations increased along the pathway gradually with the GAr, a dephosphorylated substrate of GART at 5 mmol/mol of creatinine, the FGAr at 40 mmol/mol of creatinine, the AIr at 100 mmol/mol of creatinine and the CAIr at 40 mmol/mol of creatinine. The concentrations of SAICAr, AICAr and FAICAr that are generated from the AIR, a PAICS reaction product, were increased only slightly at a range of 1 to 3 mmol/mol of creatinine (Fig. 1). The presence and concentration of AIr and CAIr in urine was unaffected by sample storage at −80 °C and five freeze-thaw cycles. Their concentrations however decreased upon common sample storage conditions with 40% of the original amount detected after thirty days at −20 °C, 40% after seven days at 4 °C, and 40% or 5% after one- or three-days storage at room temperature.

As in our previous study [5] we did not find any abnormalities in presence and/or concentrations of DNPS intermediates in the cell lysate or the media of the patient skin fibroblasts.

### Structural effects of identified PAICS variants

**Fig. 3:**
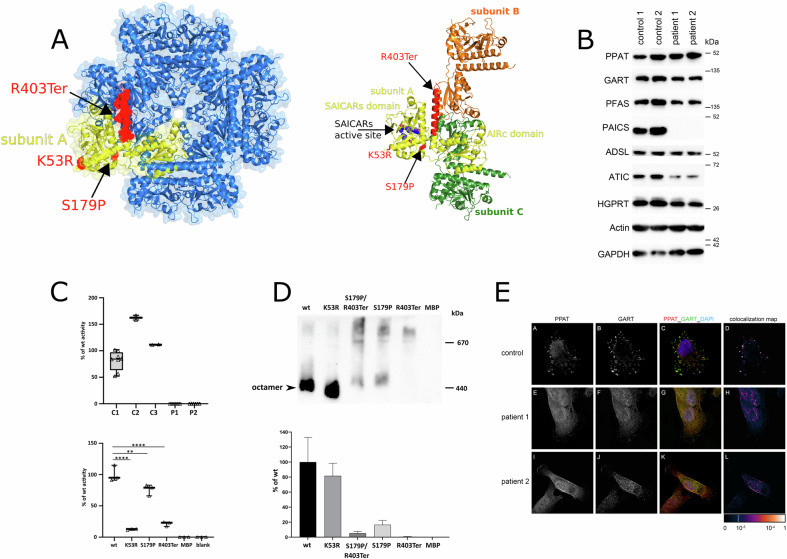
Characterization of PAICS variants: structural, biochemical, and functional analyses in fibroblasts and recombinant proteins. **A** **Structural positioning of variants in PAICS** (**PDB ID 2H31)**. On the left is octameric assembly of PAICS with one subunit highlighted in yellow. The positions of the identified variants are shown in red. On the right, a close-up view shows three subunits of PAICS octamer in different colors. SAICARs active site is highlighted by spheres. Positions of variants are highlighted in red color. **B** **Western blot analysis in skin fibroblasts** with antibodies against PPAT, GART, PFAS, PAICS, ADSL, ATIC, and HGPRT revealed a decrease in PAICS, ATIC and PFAS content, while the content of PPAT was increased. The content of other DNPS enzymes was similar to that in control fibroblasts. Protein amounts were normalized to GAPDH and actin. **C** **PAICS enzyme activities in fibroblasts and recombinant proteins**. PAICS enzyme activity was not detectable in skin fibroblasts compared to controls. Enzyme activity of mutated maltose binding-PAICS fusion proteins MBP-PAICS_S179P, MBP-PAICS_R403Ter and MBP-PAICS_K53R were reduced compared to the wild type to 76, 21 and 12%, respectively. C1-3 denote control skin fibroblast samples, while P1-2 represent patient samples. MBP-PAICS fusion proteins are indicated by their respective variant abbreviations. The MBP represents activity of the recombinant maltose binding protein devoid of PAICS. Standardized boxplot graphs with box from the first to third quartiles, whiskers to minimum and maximum, all displayed points, and the median represented by a line. The experiments were performed at least three times (*n* ≥ 3). One-way ANNOVA statistical tests were calculated by GraphPad software and *p-values* are shown: ** for *P* ≤ 0.01 and **** for *P* ≤ 0.0001. **D** **Blue native electrophoresis of PAICS**. Recombinant maltose binding-PAICS fusion proteins were expressed in *E.coli*, purified on amylose resin and cleaved with Factor Xa protease. Products of cleavage were separated on BN-PAGE, blotted and immunodetected with anti-PAICS antibodies. The wild type forms two high molecular weight structures. Both PAICS variants, Ser179Pro and Arg403Ter, individually as well as in mixture, exhibit significantly reduced octamer formation. The graph shows the relative amounts of PAICS octamer in each variant compared to the wild type (wt) (*n* = 3). PAICS protein variants are indicated by their respective abbreviations. The MBP represents immunodetection signal of the recombinant maltose binding protein devoid of PAICS. **E** **Immunofluorescent detection of PPAT and GART in skin fibroblasts**. Patients and control skin fibroblasts were cultured in purine-depleted medium and in both cases PPAT co-localized with GART in finely granular cytoplasmic structures that are characteristic of purinosome bodies. Colocalization values are converted to pseudo color, with the scale displayed in a corresponding lookup table (LUT) at the bottom right.

To assess the effects of identified variants on protein structure and enzyme assembly, we examined the crystal structure of PAICS (PDB ID 2H31). Protein subunits, each composed of AIRc and SAICARs domain, are assembled into compact homooctameric complex. The p. Ser179 residue is localized at the helix α-4 which forms a part of SAICARs domain and is positioned relatively close to oligomezation area (Fig. 3A). Replacement of the serine by proline in this position likely causes steric distortion of the surrounding helical structure. These local effects could propagate to the proximal regions including SAICARs catalytic site and oligomerization interface. This can affect stability, activity and/or octameric assembly of the enzyme. The variant p.Arg403Ter leads to deletion of the C-terminal helix. This segment forms a linker between AIRc and SAICARs domains and is critical for interactions at the oligomerization interface (Fig. 3A) [16]. The loss of this C-terminal segment may thus destabilize octameric assembly of the enzyme and lead to aberrant communication between AIRc and SAICARs domains.

To assess the effect of the respective variants on protein stability, we further utilized Rosetta Online Server. Both novel variants (Ser179Pro and Arg403Ter) exhibited significant structural destabilization, in contrary to previously described variant Lys53Arg (Supplementary Table S3). This finding suggests impaired stability of Ser179Pro and Arg403Ter variants and catalytic incompetence of the Lys53Arg variant.

### Effects of variants on PAICS enzyme and purinosome formation

In accordance with the structural prediction, we did not immunodetect PAICS in lysates of patient skin fibroblasts. Absence of PAICS was accompanied by increased amount of PPAT, decreased amounts of PFAS and ATIC (Fig. 3B) and undetectable PAICS activity (Fig. 3C). Similarly, we did not detect PAICS and its activity in lysate of patients PBMCs (data not shown). The recombinant enzyme proteins with the Ser179Pro and Arg403Ter variants showed decreased activities to 76 and 21%, respectively (Fig. 3C). Blue native electrophoresis revealed that the wt protein forms predicted octamer complex and unknown higher molecular weight structure. Both variants, individually as well as in mixture, exhibit significantly reduced octamer formation (Fig. 3D). In our previous studies we have shown that structural variants or a loss of DNPS enzymes affect purinosome assembly [5]. To this end, we cultured patient skin fibroblasts in purine-depleted media and performed parallel immunolabeling of two pairs of DNPS proteins: PPAT with GART (Fig. 3E) and ADSL with GART (data not shown). Confocal microscopy revealed that in both combinations the corresponding proteins were present with staining intensities comparable with control fibroblasts and co-localized in fine granular structures that are characteristic for purinosome formation.

## Discussion

In this work, we identified and characterized two siblings from Taiwan with PAICS deficiency who were born at term after an uncomplicated pregnancy and delivery and presented later in life with progressive cerebral atrophy, early-onset epileptic encephalopathy, psychomotor retardation, and retinopathy. The clinical presentation was less severe than the two other siblings with PAICS deficiency identified recently in a family from the Faroe Islands [5]. These infants presented prenatally with polyhydramnios, intrauterine growth retardation, severe multiple malformations, skeletal abnormalities, craniofacial dysmorphism, choanal atresia, pulmonary hypoplasia, esophageal atresia, and genitourinary abnormalities. Both infants had downhill course with progressive hypotension and hypoxia and died within 3 days of life.

The differences in clinical presentation suggest that PAICS deficiency may be phenotypically variable with a fatal neonatal form, severe postnatal form and potentially also with a moderate or mild form, as found in the ADSL deficiency [22].

The phenotypic variability in ADSL deficiency is mainly determined by the structural stability and residual catalytic capacity of the corresponding mutant ADSL protein complexes, which is also a prerequisite for the formation, stability and synthetic capacity of the purinosome [23].

To this end, in cultured skin fibroblasts, the Taiwanese patients exhibited undetectable levels of PAICS protein amounts and enzyme activity but with purinosome-like structures present. The Faroe Island patients with fatal neonatal form had normal PAICS protein amounts with enzyme activity reduced to 10%, with purinosome-like structures not detected [5]. This correlates with findings on the characteristics of recombinant proteins where the Ser179Pro and Arg403Ter proteins of Taiwanese patients had residual enzyme activities decreased to 76 and 21%, respectively, compared to 10% of the Lys35Arg protein of Faroe Island patients. In addition, the Ser179Pro and Arg403Ter variants, either individually as well as in mixture, form significantly reduced octamer complex. Therefore, the purinosome formation in skin fibroblasts with higher residual activity of corresponding recombinant mutant proteins seem to be correlated with a relatively less severe phenotype in Taiwanese patients compared with fatal neonatal form of the disease in Faroe Island patients. Alternatively or in parallel, it has been demonstrated in zebrafish that *paics* variants causes errors in vertebrate embryonic development; and that the more severe developmental phenotype is determined by maternally provided allele [24]. To this end, the maternal allele in the Taiwanese family encodes the Arg403Ter protein with a two-fold higher residual activity compared to the Lys35Arg protein in Faroe Island patients.

In this work, we also had unique opportunity to analyze for the first time the presence of the DNPS intermediates in clinical specimens of patients with PAICS deficiency. We found that the presence of AIr and CAIr, the dephosphorylated substrates of PAICS enzyme, in plasma and urine are great diagnostics markers for PAICS deficiency. We showed that under standard handling conditions both of these metabolites are stable in plasma and urine and the quantitative LC-MS/MS methodology is usable as a cost-effective diagnostic method for differential diagnosis of neurodevelopmental disorders and functional characterization of PAICS variants that may be identified via targeted or genome-wide sequencing studies.

Interestingly, we also observed slightly elevated metabolites downstream of PAICS, likely due to an increased flux through DNPS. Since a complete enzyme block would be incompatible with life, some residual flux must remain to allow for the continued production of purines. This flux is likely upregulated as a result of the reduced PAICS efficiency, which leads to higher demands of purine metabolites in the cells.

In conclusion, we have identified and characterized the second reported case of PAICS deficiency. Our observation expands the clinical spectrum of PAICS deficiency from recurrent abortions and fatal neonatal form to later onset neurodevelopmental disorders. The lack of detection and rarity of this condition may be due to poor clinical recognition and limited access to specialized laboratory tests that are diagnostic for PAICS deficiency.

## Supplementary information

Supplementary Tables

## Data Availability

The data that support the findings of this work are available on request from the corresponding author (MZ). The data are not publicly available due to privacy/ethical restrictions. The pathogenic variants identified in this work and their clinical association have been submitted to ClinVar (Submission ID: SUB14531781).
