# Supplementary material for: Expanding clinical spectrum of PAICS deficiency: Comprehensive analysis of two sibling cases
Source: Eur J Hum Genet. 2024 Nov 27;33(7):870–7. doi: 10.1038/s41431-024-01752-2 (PMC12229671; doi:10.1038/s41431-024-01752-2)
Supplement: Supplementary file 1 — Supplementary Tables [file 41431_2024_1752_MOESM1_ESM.pdf]

Table S1. Selected reaction monitoring (SRM) of purine metabolites

metabolite	Q1	Q3
**GAR**	287,1	**75,1**
	287,1	99
**GAr**	207,1	**75,1**
	207,1	171
**$^{13}C_2$-GAr**	29,1	**77,1**
**$^{13}C_2$,^{15}N-GAr**	210,1	**78,1**
**FGAR**	315,1	**103**
	315,1	114
**FGAr**	235,1	**103,1**
	235,1	75
**$^{13}C_2$,^{15}N-FGAr**	238,1	**106,1**
**FGAM** (ribotide)	314,2	**102,1**
	314,2	127
**FGAM** riboside	234,1	**102,1**
	234,1	127,0
**AIR**	296,2	**84**
	296,2	94
**AIr**	216,2	**84**
	216,2	94
**$^{13}C_2$,$^{15}N_2$-AIr**	220,2	**88**
**CAIR**	340,2	**128,2**
	340,2	110,2
**CAIr**	260,2	**128,2**
	260,2	110,2
**SAICAR**	455,2	**243,0**
	455,2	321,0
**SAICAr**	375,2	**243,0**
	375,2	321,0
**$^{13}C_4$-SAICAr**	379,2	**247,0**
**AICAR**	339,2	**110,2**
	339,2	127,2
**AICAr**	259,2	**127,2**
	259,2	110,2
**$^{13}C_2$,^{15}N-AICAr**	262,2	**130,2**
	262,2	113,2
**FAICAR**	367,1	**155,1**
	367,1	138,1
**FAICAr**	287,2	**138,2**
	287,2	155,2
**IMP**	349,1	**137,0**
	349,1	97,1
**inosine**	269,0	**137,0**
	269,0	109,9

**xanthine**	153,1	**110,1**
	153,1	136
**hypoxanthine (HX)**	137,1	**110,1**
	137,1	119,0
**$^{13}C_5$-HX**	142,1	**114,1**
**SAdo**	384,2	**252,2**
	384,2	234,1
**$^{13}C_4$-Sado**	388,2	**256,2**
**SAMP**	464,1	**251,7**
	464,1	162
**uric acid (UA)**	169,3	**141,0**
	169,2	126,1
**$^{15}N_2$-UA**	171,3	**143**

Table S2. Summary of clinical presentation of PAICS deficiency cases

	Family 1[a]	Family 2	
		Case 1	Case 2
Variants	c.158A>G p.(Lys53Arg)<br>c.158A>G p.(Lys53Arg)	c.535T>C (p.Ser179Pro)<br>c.1207C>T (p.Arg403Ter)	c.535T>C (p.Ser179Pro)<br>c.1207C>T (p.Arg403Ter)
Growth retardation	+ (intrauterine)	-	-
Polyhydramnios	+	-	-
Brachycephaly	+	+	+
Microcephaly	n.a.	+	+
Short neck	+	+	+
Flat face	+	+	+
Hypertelorism	+/-	-	-
Low-set ears	+	-	-
Poorly modulated ears	+	-	-
Macular dystrophy	n.a.	+	+
Strabismus/nystagmus	n.a.	+	+
Small nose	+	+	+
Low nasal bridge	+	+	+
Anteverted nostrils	+	+	+/-
Choanal atresia	+	-	-
Thin upper lip	n.a.	-	+
Long smooth philtrum	n.a.	Long philtrum, not smooth	-
Pulmonary hypoplasia/malformation	+	-	-
Esophagus atresia	+	-	-
Genitourinary abnormalities	Micropenis, subcoronal hypospadias	n.a.	-
Multiple skeletal malformations	+++	n.a.	-
Cognitive difficulties/encephalopathy	n.a.	+	+

n.a. - not available

[a] *Pelet A, Skopova V, Steuerwald U, Baresova V, Zarhrate M, Plaza JM, et al. PAICS deficiency, a new defect of de novo purine synthesis resulting in multiple congenital anomalies and fatal outcome. Human Molecular Genetics. 2019;28(22):3805-14.*

Table S3. Structural stability of PAICS variants determined by Rosetta scoring function.

Protein	Rosetta score	Δ WT	Impact
WT	-2415.103		
Lys53Arg	-2487.74	-72.637	more stable
Ser179Pro	-2340.91	74.193	less stable
Arg403Ter	-1683.099	732.004	dramatically less stable
